# Comparative Microbiome Analysis of a Fusarium Wilt Suppressive Soil and a Fusarium Wilt Conducive Soil From the Châteaurenard Region

**DOI:** 10.3389/fmicb.2018.00568

**Published:** 2018-04-04

**Authors:** Katarzyna Siegel-Hertz, Véronique Edel-Hermann, Emilie Chapelle, Sébastien Terrat, Jos M. Raaijmakers, Christian Steinberg

**Affiliations:** ^1^Agroécologie, AgroSup Dijon, Institut National de la Recherche Agronomique, Université Bourgogne Franche-Comté, Dijon, France; ^2^Laboratory of Phytopathology, Wageningen University, Wageningen, Netherlands; ^3^Department of Microbial Ecology, Netherlands Institute of Ecology (NIOO-KNAW), Wageningen, Netherlands

**Keywords:** bacterial community, fungal community, diversity, metabarcoding, 454 pyrosequencing, biocontrol agent

## Abstract

Disease-suppressive soils are soils in which specific soil-borne plant pathogens cause only limited disease although the pathogen and susceptible host plants are both present. Suppressiveness is in most cases of microbial origin. We conducted a comparative metabarcoding analysis of the taxonomic diversity of fungal and bacterial communities from suppressive and non-suppressive (conducive) soils as regards Fusarium wilts sampled from the Châteaurenard region (France). Bioassays based on Fusarium wilt of flax confirmed that disease incidence was significantly lower in the suppressive soil than in the conducive soil. Furthermore, we succeeded in partly transferring Fusarium wilt-suppressiveness to the conducive soil by mixing 10% (w/w) of the suppressive soil into the conducive soil. Fungal diversity differed significantly between the suppressive and conducive soils. Among dominant fungal operational taxonomic units (OTUs) affiliated to known genera, 17 OTUs were detected exclusively in the suppressive soil. These OTUs were assigned to the *Acremonium, Chaetomium, Cladosporium, Clonostachys, Fusarium, Ceratobasidium, Mortierella, Penicillium, Scytalidium*, and *Verticillium* genera. Additionally, the relative abundance of specific members of the bacterial community was significantly higher in the suppressive and mixed soils than in the conducive soil. OTUs found more abundant in Fusarium wilt-suppressive soils were affiliated to the bacterial genera *Adhaeribacter, Massilia, Microvirga, Rhizobium, Rhizobacter, Arthrobacter, Amycolatopsis, Rubrobacter, Paenibacillus, Stenotrophomonas*, and *Geobacter*. Several of the fungal and bacterial genera detected exclusively or more abundantly in the Fusarium wilt-suppressive soil included genera known for their activity against *F. oxysporum*. Overall, this study supports the potential role of known fungal and bacterial genera in Fusarium wilt suppressive soils from Châteaurenard and pinpoints new bacterial and fungal genera for their putative role in Fusarium wilt suppressiveness.

## Introduction

Plant diseases caused by soil-borne microorganisms, including fungi, oomycetes, bacteria, nematodes as well as subterranean insects, regularly result in extensive losses in agricultural production every year. Because of its role in many enzymatic activities governing the functioning of the soil, particularly in biogeochemical cycles, reorganization, and mineralization of organic matter, and feeding of plants, soil microbial diversity is a determining component of soil health (Garbeva et al., 2004; Janvier et al., 2007; Chaparro et al., 2012; Larkin, 2015; van Bruggen et al., 2015). Hence, growth of plant pathogens in soils and subsequent plant infection can be reduced through competitive interactions with the soil microbial community which results in defining disease suppressive soils (Weller et al., 2002; Raaijmakers et al., 2009; Lundgren and Fergen, 2011; Kyselková and Moënne-Loccoz, 2012; Penton et al., 2014). Indeed, soils in which disease incidence or severity commonly remain low in spite of the presence of the pathogen, a susceptible host plant and climatic conditions that would allow disease development are called disease suppressive soils (Baker and Cook, 1974). General disease suppressiveness of soils is based on multitrophic interactions and can be modulated by soil management practices that affect total microbial activity (Mazzola and Gu, 2002; Stirling et al., 2012). In addition to general suppressiveness, some soils exhibit an additional level of suppressiveness targeted to a specific soil-borne plant pathogen. Specific suppressiveness is attributed to the converging activities of specific members of the soil microbial community that interfere with the disease cycle of the pathogen. This is the case, for example, of bacteria in the *Pseudomonas* group that produce metabolites such as pyoverdins, iron-chelating siderophores, and make iron difficult to access for the pathogenic fungus *F. oxysporum*, this mechanism being added to the competition for carbon to which this same pathogen is confronted (Lemanceau et al., 1993; Alabouvette, 1999). Specific suppressiveness can be eliminated by soil sterilization, steam pasteurization, or gamma-irradiation and can be transferred by mixing a small amount of natural suppressive soil into both previously disinfected suppressive soil and natural conducive soil (Alabouvette, 1986; Weller et al., 2002; Garbeva et al., 2004; Mendes et al., 2011). Although the role of abiotic components cannot be ruled out (Amir and Alabouvette, 1993; Almario et al., 2013a), all of these demonstrations clearly indicate that a focus must be placed on the biotic component (Alabouvette, 1999).

This intriguing phenomenon of specific disease suppressiveness has been described for a number of soil-borne pathogens, including bacteria, fungi, oomycetes, and nematodes. Among fungi, special attention has been paid to soils suppressive to take-all disease of wheat (Weller et al., 2002), damping-off diseases caused by *Rhizoctonia solani* (Mendes et al., 2011) and wilt diseases caused by different formae speciales of *Fusarium oxysporum* (Toussoun, 1975; Louvet et al., 1976; Alabouvette, 1986; Alabouvette et al., 2009). For decades, many vegetables were and are still produced in the market gardening region of Châteaurenard, in France and yet very few symptoms of Fusarium wilt are observed despite the presence of *F. oxysporum* in the soil which led to consider this soil as suppressive to this disease (Louvet et al., 1976; Alabouvette, 1986). Because the situation was unique, we conducted various studies, over the past 40 years, to determine the microorganisms and mechanisms involved in Fusarium wilt suppressiveness of this soil from the Châteaurenard region. Alabouvette (1986) postulated that the suppressive nature of this soil relied on both (i) a general mechanism of competition for nutrients caused by the whole soil microflora and (ii) a specific competition between pathogenic and non-pathogenic *Fusarium* strains. Subsequent studies further revealed that bacteria of the *Pseudomonas* genus contributed, in combination with non-pathogen *F. oxysporum*, to suppressiveness via siderophore-mediated competition for iron and via the production of antifungal phenazines (Lemanceau and Alabouvette, 1993; Duijff et al., 1999; Mazurier et al., 2009). We used next-generation sequencing to conduct a comparative metabarcoding analysis of the microbiomes of both the Fusarium wilt suppressive soil of Châteaurenard and a non-suppressive soil from a nearby field. The analysis was based on 454-pyrosequencing of the fungal internal transcribed spacer (ITS) region and the bacterial 16S rRNA gene. The aim was to detect and identify fungal and bacterial genera associated with Fusarium-wilt-suppressive soils not yet identified and get new insights into potentially novel microbial genera and mechanisms involved in Fusarium wilt suppressiveness.

## Materials and methods

### Soil sampling

The Fusarium-wilt-suppressive soil was harvested from a field that had remained a fallow for several years in the Châteaurenard region, France (43°53′15″N, 4°51′36″E). The conducive soil was sampled nearby from a temporarily uncultivated field following muskmelon cropping and before lettuce cropping (43°51′53″N, 4°50′04″E). The two soils were geographically very close (3 km apart). Both were collected in October 2011, air-dried and sieved to 4 mm. Both were loamy soils. Physicochemical profiles were respectively 17.1% clay, 47.4% silt, 35.5% sand, 1.98% organic matter (OM), 0.9 N (g/kg), and pH 8.5 for the suppressive soil, and 20.6% clay, 43.2% silt, 36.2% sand, 4.6% OM, 1.5 N (g/kg), pH 8.06 for the conducive soil. Differences in organic matter content, nitrogen content and pH were related to the current uses of the soils. To confirm the biological nature of the soil suppressiveness, the suppressive soil was autoclaved three times on three consecutive days for 20 min at 120°C and stored at room temperature for 1 week before use.

### Assessment of soil disease suppressiveness

Soil disease suppressiveness was tested under greenhouse conditions with flax as the host plant and the flax pathogen *F. oxysporum* f. sp. *lini* MIAE00347 (Collection of Microorganisms of Interest for Agriculture and Environment, INRA Dijon, France). The plants were grown in different types of soil: suppressive soil (S), conducive soil (C), conducive soil amended with 10% (w/w) of suppressive soil (referred to as mixed soil or M), and heat-treated suppressive soil. Soils were inoculated with 10^3^ conidia of the pathogen per mL of soil, and flax (*Linum usitatissimum* L. variety Opaline) seeds were sown on the same day. For each modality, three replicates were performed with 20 plants/replicate. Non-inoculated soils were used as controls. Plants were grown in a growth chamber with 70% relative humidity, a 16/8 h daylight/dark photoperiod at 17°C (day) and 15°C (night) the first week, and 25°C (day) and 22°C (night) afterwards. Diseased plants were recorded 25 days after inoculation. Data were subjected to one-way analysis of variance (ANOVA). Pairwise comparisons were performed using Fisher's test (*P* < 0.05).

### Rhizospheric soil sampling

Flax seeds were sown in 9/9/9.5 cm pots containing 300 g of soil. Twenty plants per pot were grown under the same conditions of light and humidity as above. Four modalities were prepared: soil S, soil C, soil M, and suppressive soil inoculated with 10^3^ conidia of *F. oxysporum* f. sp. *lini* MIAE00347 per mL of soil (referred to as pathogen-inoculated suppressive soil or IS soil). The inoculum dose used was relatively low (10^3^ conidia per mL) to ensure that plants do not die too quickly in the conducive soil and prevent to collect the active rhizospheric soil needed to conduct microbial communities comparative analyses. Three replicates per modality were performed. After 25 days, the rhizospheric soil was collected from each replicate. The root systems were isolated and shaken to remove free soil particles. The soil around the roots influenced by root development and plant exudates was considered as rhizospheric soil. It was sieved to 2 mm to remove fine roots and organic debris. Large pieces of roots were removed manually with tweezers. Two grams of soil samples from each modality were placed in 2-ml cryotubes and frozen at −20°C for further DNA extractions.

### DNA extraction, PCR amplification, and construction of pyrosequencing libraries

The protocol used for DNA extractions was described by Plassart et al. (2012). Extraction was performed by mechanical lysis using FastPrep®-24 (MP-Biomedicals, NY, USA) with a lysis buffer and two purification steps with PVPP (polyvinyl polypyrrolydone) minicolumns (BIORAD, Marne-la-Coquette, France) and Geneclean Turbo kit (Q-Biogene, Illkirch, France). Total DNA was extracted from each replicate of the four modalities. Each purified DNA sample was quantified by a fluorometric assay using the PicoGreen dsDNA Assay Kit (Invitrogen Life Technologies) and the StepOnePlus™System (Applied Biosystems®) according to the manufacturer's instructions. DNA extracted from each soil sample served as a template in PCR reactions to amplify a fungal barcode and a bacterial barcode. Three replicates were used for each of the four soil modalities. PCR reactions were performed in quadruplicate for each replicate, with 20 ng of soil DNA.

For fungal identification, the variable internal transcribed spacer 1 (ITS1) region was amplified using specific fungal primers ITS1F (5′-CTTGGTCATTTAGAGGAAGTAA-3′) and ITS2 (5′-GCTGCGTTCTTCATCGATGC-3′; White et al., 1990; Gardes and Bruns, 1993; Buée et al., 2009). The primers were tagged with four-base-pair multiplex identifiers (MIDs) at the 5′ and 3′ positions to specifically identify each sample, as recommended by the manufacturer (Beckman Coulter Genomics). PCR conditions were as follows: 95°C for 5 min, 35 cycles of 30 s at 95°C, 40 s at 53°C, and 45 s at 72°C, followed by 7 min at 72°C.

For bacterial identification, a 16S rRNA gene fragment (partial V3, V4 and partial V5) was amplified using primers 530F (5′-ACTCCTACGGGAGGCAGCAG-3′; Acosta-Martínez et al., 2008) and 803R (5′-CTACCNGGGTATCTAAT-3′; Zancarini et al., 2013). Ten-base-pair MIDs at the 5′ and 3′ positions were added to the primers to specifically identify each sample, as recommended by the manufacturer (Beckman Coulter Genomics). PCR conditions were as follows: 95°C for 10 min, 30 cycles of 30 s at 95°C, 30 s at 62°C, and 60 s at 72°C, followed by 10 min at 72°C. All amplifications were performed in a Mastercycler (Eppendorf, Hamburg, Germany). For each sample, amplicons of the four replicated PCRs were pooled and purified using a MinElute PCR Purification Kit (Qiagen, Courtaboeuf, France) following the manufacturer's protocol. Amplicon concentrations were then estimated by fluorometric assay (PicoGreen dsDNA Assay Kit). For each of the two amplified barcodes, an equimolar pooling of all samples was prepared (total DNA amount: ~3.2 μg per library). Adapter sequences were added by ligation as recommended by the manufacturer, and 454-pyrosequencing was carried out by Beckman Coulter Genomics (Danvers, USA) on a Genome Sequencer FLX 454 (Life Sciences/Roche Applied Biosystems).

### Bioinformatics analysis

Concerning the sequenced ITS1 region, sequences were sorted into different files according to their MID using the sfffile program of Roche 454 main software with default parameters. Mismatches were not allowed for MIDs and were removed. Sequences were then cleaned by trimming and denoising using trim.flows in Mothur version 1.20.1 with the default parameters (Schloss et al., 2009), and reverse-complemented if needed. ITS1 was extracted using Fungal ITS Extractor, version 2 (Nilsson et al., 2010) and sequences were filtered based on a minimal length of 100 bp. Operational taxonomic units (OTUs) were generated after two successive clustering steps using Uclust version 3.0 [Usearch version 5.2.32, (Edgar, 2010)] at 97% similarity. The first clustering included all sequences, and the second clustering was conducted with the batch of consensus sequences from the previous clustering.

Concerning the sequenced 16S region, sequences were analyzed using the GnS-PIPE pipeline initially developed by the GenoSol platform (INRA, Dijon, France) and recently optimized (Terrat et al., 2012, 2015). First, all the raw 16S reads were sorted according to their multiplex identifier sequences. Then, a preprocessing step was realized to filter and delete low-quality reads based on (i) their length (<350 bases), (ii) their number of ambiguities (deletion of reads with one N or more), and (iii) their primer sequence(s) (the distal and proximal primer sequences must be complete and without errors). A PERL program was then applied for rigorous dereplication (i.e., clustering of strictly identical sequences with same length). The dereplicated reads were aligned using INFERNAL alignment (Cole et al., 2009; v1.0.2 with chosen parameters: –hbanded, –sub, –dna) to obtain a global alignment. Then, aligned sequences were clustered into OTUs at 93.8% of similarity using the CrunchClust V43 program (Hartmann et al., 2012) that groups rare reads with abundant ones and does not count differences in homopolymer lengths (default parameters were selected). Here, an OTU is defined by the most abundant read, known as the centroid, and every read in the OTU must have similarity above the given similarity threshold with the centroid). The chosen level of similarity was defined by an *in silico* approach (data not shown). We first extracted all known and reliable microbial sequences (bacteria and *archaea*) from the SILVA database (release v111) (Quast et al., 2013). We kept from these sequences only the amplified regions of sequences using our primer set, and deleted those with too many mismatches with our primer set (more than 3 mismatches with one primer). Then, all these “artificial reads” with a reliable taxonomy were clustered using our specific program of clustering at various threshold levels (100% to 90%, with a 0.1% step). Regarding the results and the amplified regions, the 93.8% threshold was the best suited to efficiently define the genus level, as it was the closest one to the genus level defined by the SILVA taxonomy of analyzed sequences.

For both the sequenced ITS and 16S regions, a filtering step was carried out to check all singletons (OTUs supported by only one sequence likely to be artifacts, such as PCR chimeras) and potentially to delete them from downstream analysis based on the quality of their taxonomic assignments. Then, to efficiently compare datasets and avoid biased community comparisons, high-quality reads were normalized by random selection: each dataset with a large number of reads was randomly cut down to the same number of reads as the dataset with the lowest number of reads (3,451 and 2,505 reads for fungal and bacterial communities, respectively) using a homemade Perl program (similar to the single_rarefaction.py with default parameters from QIIME). The retained high-quality reads were then used for taxonomy-independent analyses, and several diversity and richness indices were determined using the defined OTU composition. More precisely, we used the number of bacterial and fungal OTUs from each sample to determine Shannon and Evenness indices as indicators of soil microbial diversity and structure, respectively. Taxonomy-based analysis was also performed using similarity approaches against: (i) the UNITE database (fungal rDNA ITS sequence database; Kõljalg et al., 2013) using the Basic Local Alignment Search Tool algorithm BLASTn version 2.2.23 (Altschul et al., 1997) with the filter turned off for fungal sequences; (ii) a dedicated reference database from SILVA (Quast et al., 2013), using the USEARCH tool (Edgar, 2010) for bacterial sequences. Finally, global analysis of the soil samples was computed by merging all homogenized high-quality reads from each sample into one global file and defining OTUs as previously described before subsequent analyses.

All raw sequences collected in this study have been deposited in the European Bioinformatics Institute nucleotide sequence database system under the accession number PRJEB24081.

### Statistical analysis and heat map computation

The OTUs defined for fungal and bacterial datasets were used to perform rarefaction analysis using Analytic Rarefaction version 1.3 (Hunt Mountain Software, Department of Geology, University of Georgia, Athens, GA, USA). The differences in fungal and bacterial community compositions (number of genera, OTUs, Shannon and Evenness indices) were assessed by Kruskal–Wallis tests under XLSTAT software (Addinsoft®). The OTU tables obtained from ITS1 and 16S datasets were used to perform an ordination analysis by means of Principal Component Analysis (PCA), using ADE-4 version 2001 (Biometry and Evolutionary Biology Laboratory, University Claude Bernard Lyon 1). For each microbial community, the numbers of unique OTUs and shared OTUs among the different soils were compared in Venn diagrams using VENNY (Oliveros, 2007).

Heat maps were built by applying the heatmap.2 function implemented in “gplots” R package based on the relative abundance values of the dominant fungal OTUs (for which the relative abundance level in one soil at least was higher than 1%) and dominant bacterial OTUs (for which the relative abundance level in one soil at least was higher than 1.5%) across all samples using the global matrices. Datasets were analyzed together, so the mean values of Z-scores were from the total dataset. The relative abundance levels of each row (corresponding to each OTU) were expressed as median-centered Z-scores between all samples, and the colors scaled to standard deviations of the corresponding Z-scores. All these analyses were performed with R free software (version 3.2.3). Moreover, to minimize the probability of incorrect assignments of the dominant OTUs, taxonomic assignments of representative sequences were refined by checking taxonomic assignments of dominant fungal OTUs against the GenBank database. The default algorithm parameters were used, and environmental sequences were excluded. Only sequences associated with a publication and a description of the corresponding strain were considered. Assignments were taken into account at the genus level. Some synonyms were corrected using the online *Species Fungorum* database (Kirk, 2017). For the few OTUs that had the same percentage of similarity with several fungal genera or families, assignment was made at the family or order level, respectively. Concerning the dominant bacterial OTUs, their taxonomic assignments were checked using SINA online (Pruesse et al., 2012), the SILVA database (version 123), and default parameters. SINA uses the search result to derive a classification with the LCA (lowest common ancestor) method. Each query sequence is assigned the shared part of the classifications of the search results. OTU abundance levels in the different soils were compared based on Kruskal-Wallis tests using XLSTAT software (Addinsoft®).

## Results

### Disease suppressiveness of the soils

The plants grown in the non-inoculated soils showed no wilt disease symptoms. Typical symptoms of Fusarium wilt were observed in pathogen-inoculated soils. The percentage of healthy plants was significantly higher (*P* < 0.05) in soil S (46.6 ± 10.6) and soil M (43.5 ± 19.4) than in soil C (15.3 ± 5.7). The percentage of healthy plants was significantly lower (*P* < 0.05) in heat-treated suppressive soil (9.2 ± 0.3) than in soils S, M and C, further confirming the biological nature of the soil suppressiveness.

### Microbiome data acquisition and statistics

Fungal and bacterial targeted regions were successfully amplified by PCR and sequenced for all soils. The raw sequence libraries were filtered to remove reads originating from sequencing errors or putative chimeric sequences. For the fungal and bacterial datasets, 81,902 reads (88% of raw sequences), and 67,675 reads (89% of raw sequences) passed all quality controls, respectively. The number of high-quality reads ranged from 3,451 to 10,821 for fungal datasets, and from 2,505 to 8,842 for bacterial datasets. After homogenization, a total of 41,412 high-quality fungal ITS sequences were clustered into 1,798 OTUs for all soil samples. Among them, 570 OTUs were considered as singletons, and represented 31.7% of the detected richness. Regarding bacterial communities, 2,280 distinct OTUs, including 1,162 singletons (50.9% of the richness), were observed for 30,060 high-quality bacterial 16S rDNA sequences. Rarefaction curves were drawn for fungal and bacterial datasets based on OTUs (Supplementary Figure 1). Fungal curves showed that: (i) the number of OTUs at 97% similarity increased with the number of sequences, and saturation was not reached for all soils; (ii) based on soil replicates, however, the number of sequences was sufficient to obtain a representative coverage of the major fungal groups; and (iii) differences among soils were recorded in the slope and level of the curves (Supplementary Figure 1A). As regards bacterial richness, rarefaction curves also revealed that the number of OTUs increased with the number of reads, without reaching a plateau, and reads were in sufficient numbers to allow for an accurate description of the major bacterial genera in each of the soil samples (Supplementary Figure 1B). Interestingly, no significant differences were detected in the slope and level of bacterial curves in any of the soils, showing less heterogeneity of bacterial richness among the four modalities.

Fungal and bacterial communities were also evaluated using richness and diversity indices (Table 1). Similar numbers of genera were detected in the different soils. However, significant differences among soils were recorded (*P* < 0.05) for the number of fungal OTUs, and for Shannon and Evenness indices. Thus, soil M harbored the highest fungal richness (462.0 ± 26 OTUs on average), and soils C and IS the lowest. However, soils C and M had the highest Shannon indices, and soil IS the lowest. Finally, evenness was significantly higher in soil C (*P* < 0.05) than in soils S and IS. Contrary to fungal communities, bacterial communities did not significantly differ in the number of genera, the number of OTUs, and the Shannon and Evenness indices between the different soils (Table 1).

**Table 1:** Fungal and bacterial richness and diversity indices of the conducive soil (C), mixed soil (M), suppressive soil (S), and pathogen-inoculated suppressive soil (IS).

**Community**	**Soil**	**Number of genera**	**Number of OTUs**	**Shannon's index**	**Evenness index**
Fungi	C	69.7 (± 3) *a*	380.3 (± 29) *a*	4.32 (± 0.03) *a*	0.73 (± 0.011) *a*
	M	65.0 (± 2) *a*	462.0 (± 26) *b*	4.39 (± 0.10) *a*	0.71 (± 0.010) *a,b*
	S	68.7 (± 4) *a*	406.3 (± 19) *a,b*	4.17 (± 0.10) *a,b*	0.69 (± 0.016) *b*
	IS	71.3 (± 6) *a*	360.3 (± 16) *a*	4.01 (± 0.11) *b*	0.68 (± 0.014) *b*
Bacteria	C	260.3 (± 5) *a*	542.3 (± 22) *a*	5.10 (± 0.21) *a*	0.81 (± 0.028) *a*
	M	262.6 (± 5) *a*	568.3 (± 11) *a*	5.12 (± 0.06) *a*	0.80 (± 0.009) *a*
	S	260.0 (± 2) *a*	537.0 (± 23) *a*	5.19 (± 0.09) *a*	0.82 (± 0.013) *a*
	IS	261.3 (± 2) *a*	541.0 (± 20) *a*	5.19 (± 0.11) *a*	0.82 (± 0.014) *a*

*Means were calculated from three replicates per soil (C, M, S, and IS). Standard errors of the means are indicated in parentheses. Significant differences among soils are indicated by letters (a–a,b–b)*.

### Fungal and bacterial community composition

**Figure 1:**
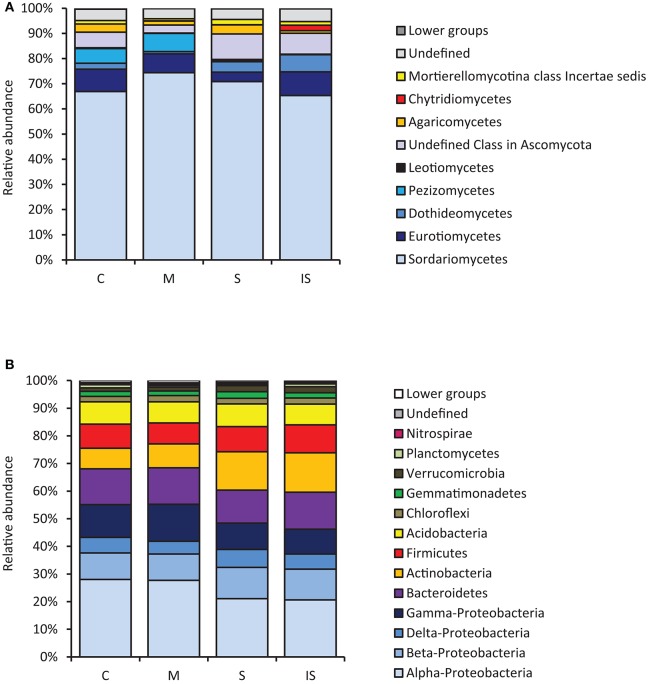
Relative abundance levels of fungal classes **(A)** and bacterial phyla **(B)**, expressed as percentages of all sequences, detected in the conducive soil (C), mixed soil (M), suppressive soil (S), and pathogen-inoculated suppressive soil (IS).

Fungal and bacterial sequences were assigned at different taxonomic levels (phylum, class, order, family, and genus; Supplementary Table 1). Concerning fungal communities, most assigned sequences belonged to the *Ascomycota* phylum (90.9%). *Basidiomycota* and *Zygomycota* accounted for 2.6 and 1.5% of the sequences, respectively. The proportion of *Chytridiomycota* and *Glomeromycota* was <1%. Finally, 4.4% of the sequences were not assigned and were considered as undefined. At the class level, *Sordariomycetes* were dominant within *Ascomycota*, followed by *Eurotiomycetes, Dothideomycetes*, and *Pezizomycetes* (Figure 1A).

Concerning bacterial communities, the soils had the same overall bacterial composition, with *Proteobacteria, Bacteroidetes, Actinobacteria, Firmicutes*, and *Acidobacteria* as major phyla (Figure 1B). *Proteobacteria* was the dominant bacterial phylum and represented 46–55% of all bacterial DNA sequences. Several minor phyla (*Chloroflexi, Gemmatimonadetes, Verrucomicrobia, Planctomycetes*, and *Nitrospirae*) were also identified; they accounted for ca. 7% of bacterial communities. However, a few differences were found among soils, with more *Gamma-Proteobacteria* in soil M than in soils S and IS (*P* < 0.05). In contrast, *Actinobacteria* were more represented in soils S and IS than in soil C (*P* < 0.05).

### Microbial community composition associated with suppressive and conducive soils

**Figure 2:**
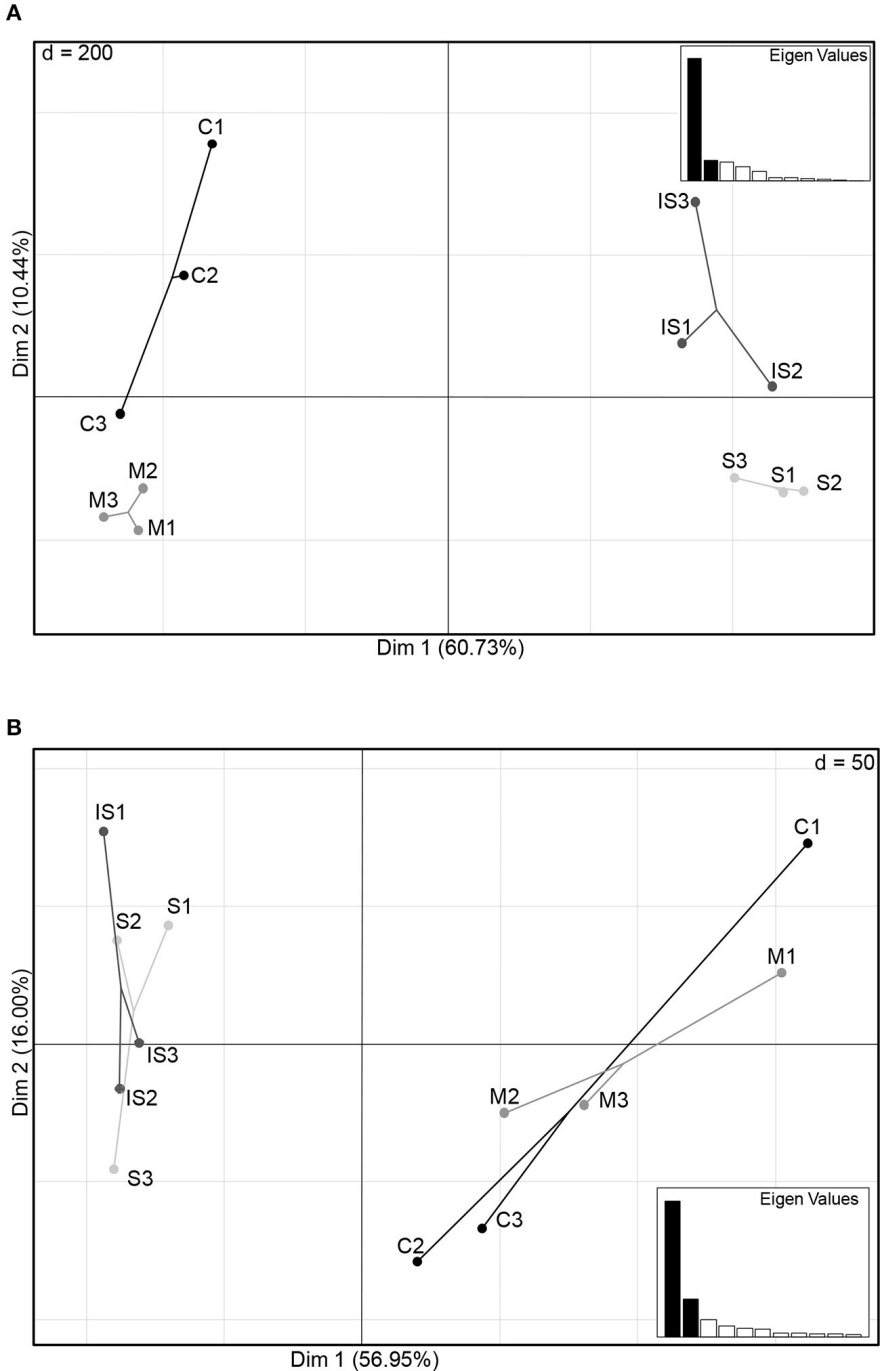
Principal component plots of the conducive soil (C), mixed soil (M), suppressive soil (S), and pathogen-inoculated suppressive soil (IS) generated from fungal **(A)** and bacterial **(B)** OTU matrices of ITS1 and 16S relative abundance levels.

The structure of the fungal and bacterial communities was analyzed by PCA to obtain an ordination on a factorial map and to compare the soils based on global OTU composition (Figure 2). A clear discrimination of fungal communities based on soil types was observed (Figure 2A). In particular, two groups of soils were separated along the first axis: soils C and M on the one hand, and soils S and IS on the other hand. Moreover, soil C grouped apart from soil M, and soil S apart from soil IS along the second axis. Concerning bacterial communities, sample discrimination was not so obvious (Figure 2B): soils C and M were separated from soils S and IS along the first axis, but no distinction of these two groups was visible along the second axis.

**Figure 3:**
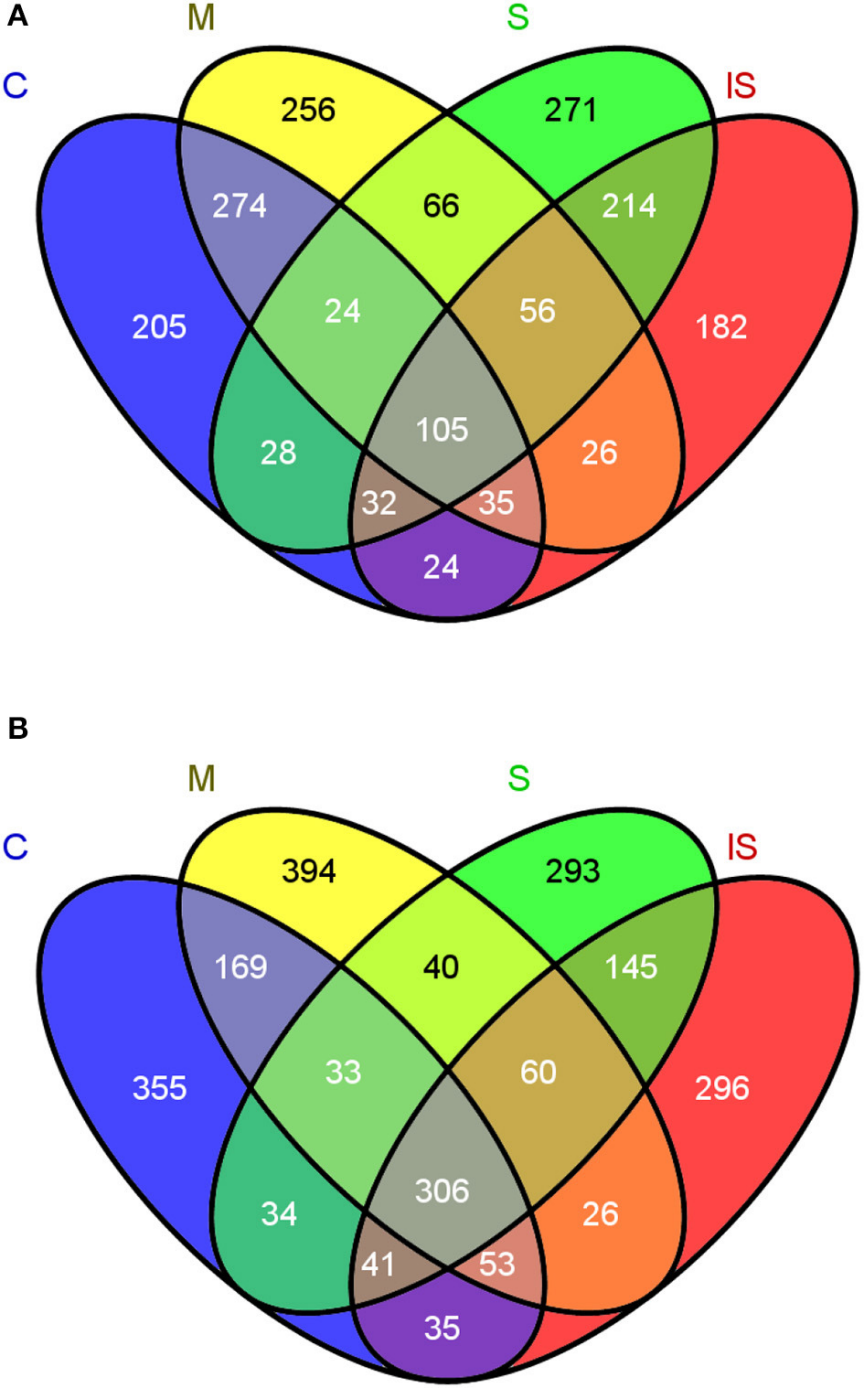
Venn diagrams indicating the numbers of shared and unique OTUs between the conducive soil (C), mixed soil (M), suppressive soil (S), and pathogen-inoculated suppressive soil (IS) in fungal **(A)** and bacterial communities **(B)**.

Regarding OTU composition, 189 fungal OTUs and 414 bacterial OTUs were shared between soils C and S (Figure 3). In the fungal community, 205 OTUs were unique in soil C, and 271 in soil S. In the bacterial community, 355 OTUs were unique in soil C, and 293 in soil S. To further focus on the microbial genera associated with disease suppressiveness, we compared the fungal and bacterial community compositions between soils S and M on the one hand, and between soils S and IS on the other hand. 122 fungal OTUs and 100 bacterial OTUs were shared between soils S and M but absent from soil C. In addition, 214 fungal OTUs and 145 bacterial OTUs shared between soils S and IS were potentially promoted by the presence of the pathogen.

**Figure 4:**
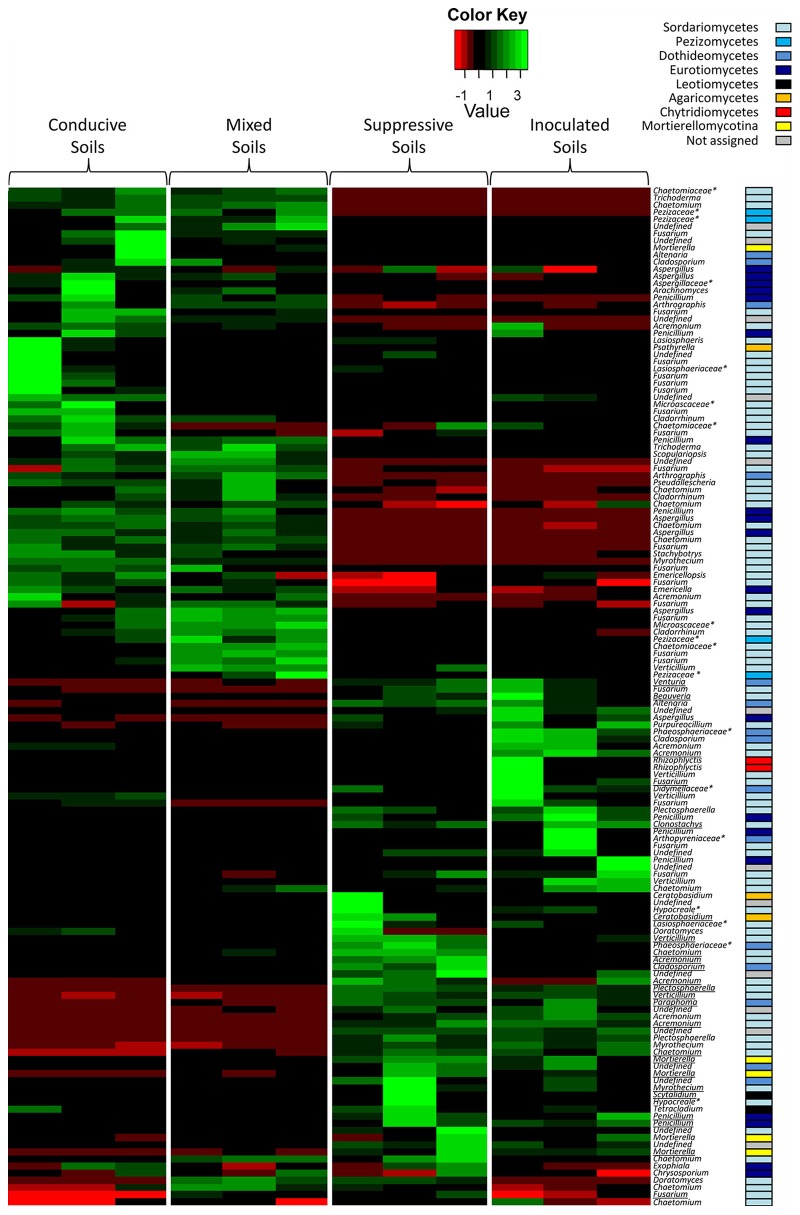
Heat map comparison of the dominant fungal genera detected in the soils according to each modality. The four different modalities (conducive soil, mixed soil, suppressive soil, and pathogen-inoculated suppressive soil) were organized based on the UPGMA dendrogram of UNIFRAC weighted and normalized distances between corresponding soil samples. The legend shows the Z-scores (relative abundance levels are expressed as median-centered Z-scores between all samples, and colors are scaled to standard deviations). OTUs with a star (^*^) indicate groups that were not assigned to a precise fungal genus, but to a higher taxonomical group. Underlined OTUs indicate significant differences in the relative abundance levels of particular fungal genera in the four modalities.

**Figure 5:**
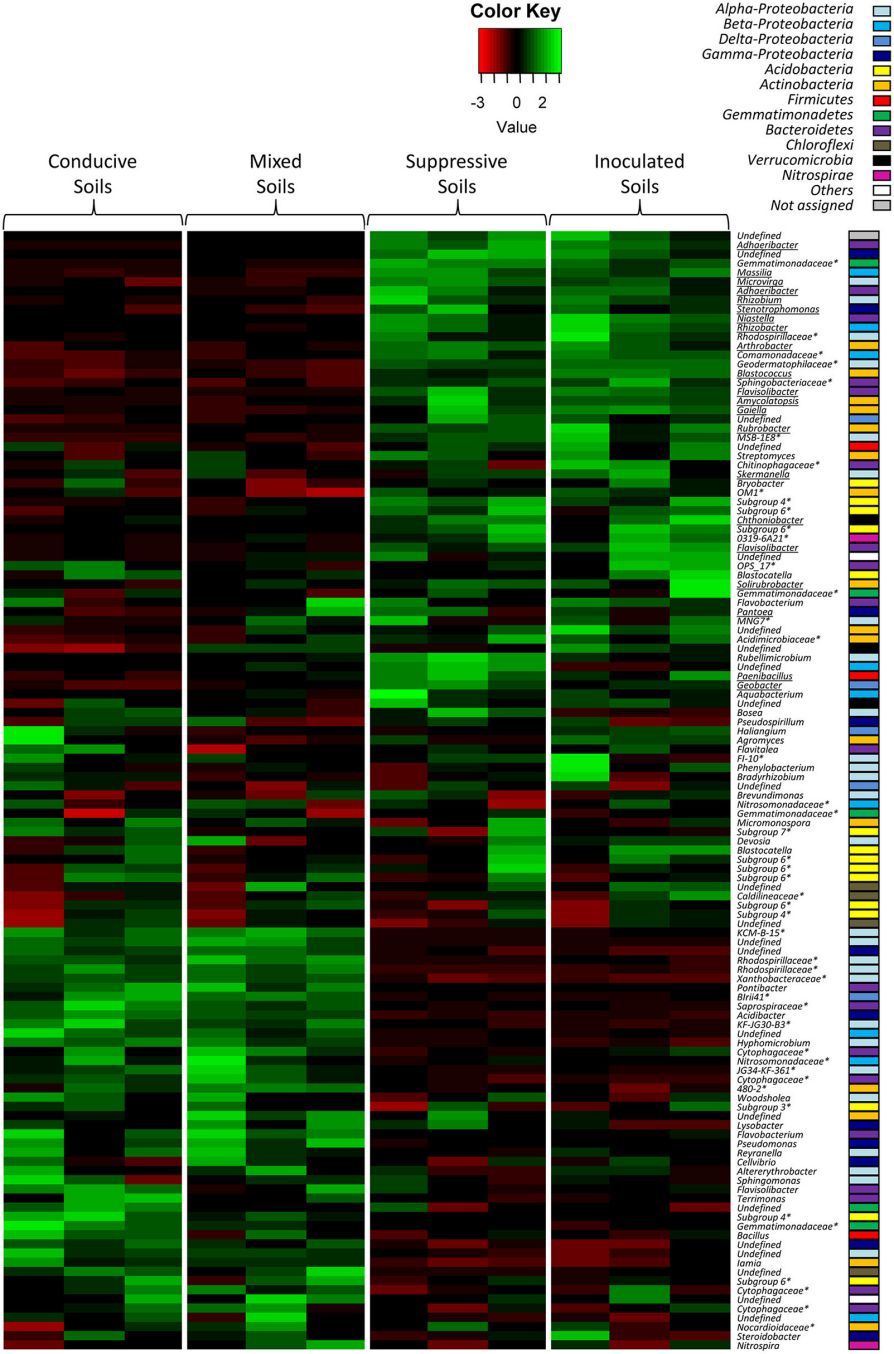
Heat map comparison of the dominant bacterial genera detected in the soils according to each modality. The four different modalities (conducive soil, mixed soil, suppressive soil, and pathogen-inoculated suppressive soil) were organized based on the UPGMA dendrogram of UNIFRAC weighted and normalized distances between corresponding soil samples. The legend shows the Z-scores (relative abundance levels are expressed as median-centered Z-scores between all samples, and colors are scaled to standard deviations). OTUs with a star (^*^) indicate groups that were not assigned to a precise bacterial genus, but to a higher taxonomical group. Underlined OTUs indicate significant differences in the relative abundance levels of particular bacterial genera in the four modalities.

To combine taxonomic assignment and soil global OTU composition, we compared microbial community compositions based on the relative abundance of the detected microbial genera using a heat map. Due to the large size of the dataset, only the most dominant fungal OTUs (relative abundance level > 1%) and bacterial OTUs (relative abundance level > 1.5%) were mapped (Figures 4, 5). The 143 dominant fungal OTUs represented in the heat map accounted for 83% of total reads on average, and the 122 dominant bacterial OTUs kept in the heat map accounted for 72% of total reads on average (Figures 4, 5). Firstly, these heat maps showed that biological replicates from a same soil were more similar to each other than replicates from other soils, whatever the soil. Moreover, evidence of clear differences between soils C and M on the one hand, and soils S and IS on the other hand was illustrated by both bacterial and fungal community compositions. Concerning fungal communities, 24 OTUs affiliated to 15 known genera were relatively more abundant in soils S and IS than in soil C (*P* < 0.05; Figure 4, underlined OTUs). Among them, 17 OTUs were not detected at all in soil C. They were assigned to the *Acremonium, Chaetomium, Cladosporium, Clonostachys, Fusarium, Ceratobasidium, Mortierela, Penicillium, Scytalidium*, and *Verticillium* genera. Interestingly, these OTUs were also detected in soil M. All 10 genera mentioned above, exclusively detected in soils S, IS and M, have been described for their antagonistic activity against diverse plant pathogens (Table 2). Moreover, the *Acremonium, Chaetomium, Clonostachys, Fusarium*, and *Penicillium* genera include strains used in the biological control of pathogenic *F. oxysporum* (Table 2).

**Table 2:** Microbial genera including OTUs associated with suppressive soil (S), pathogen-inoculated suppressive soil (IS), and mixed soil (M) and known to include biological control agents against fungal diseases.

**Microorganism**	**Genus**	**Biocontrol agent**	**Target pathogens**	**References**
Fungi	*Acremonium*	*Acremonium* sp.	*Fusarium oxysporum* f. sp. *melonis, Oidium* spp.	Mmbaga et al., 2008; Suarez-Estrella et al., 2013.
	*Ceratobasidium*	*Ceratobasidium* sp.	*Rhizoctonia solani*	Mosquera-Espinosa et al., 2013.
	*Chaetomium*	*Chaetomium cupreum, C. globosum*	*Fusarium oxysporum* f. sp. *lycopersici, Phytophthora palmivora, P. parasitica, P. cactorum, Sclerotium rolfsii, Setosphaeria turcica*	Soytong et al., 2001; Zhang et al., 2013; Hung et al., 2015.
	*Cladosporium*	*Cladosporium tenuissimum, Cladosporium* sp.	*Cronartium flaccidum, Peridermium pini, Oidium* spp.	Moricca et al., 2001; Mmbaga et al., 2008.
	*Clonostachys*	*Clonostachys rosea*	*Botrytis cinerea, Fusarium graminearum, F. oxysporum, Rhizoctonia solani*	Dubey et al., 2014; Tian et al., 2014; Borges et al., 2015.
	*Fusarium*	*Fusarium oxysporum*	Pathogenic *F. oxysporum*	Alabouvette et al., 2009.
	*Mortierella*	*Mortierella* sp.	*Verticillium dahliae*	Alstrom, 2000.
	*Penicillium*	*Penicillium oxalicum, Penicillium* sp.	*Fusarium oxysporum* f. sp. *lycopersici, Rhizoctonia solani, Oidium* spp.	De Cal et al., 2000; Larena et al., 2003; Nicoletti et al., 2004; Mmbaga et al., 2008.
	*Scytalidium*	*Scytalidium uredinicola*	*Endocronartium harknessii*	Moltzan et al., 2001.
	*Verticillium*	*Verticillium biguttatum*	*Rhizoctonia solani*	McQuilken and Gemmell, 2004; Tsror, 2010.
Bacteria	*Arthrobacter*	*Arthrobacter koreensis, A. luteolus, A. gandensis*	*Xanthomonas axonopodis* pv. *passiflorae*	Halfeld-Vieira et al., 2015.
	*Paenibacillus*	*Paenibacillus ehimensis*	*Fusarium oxysporum* f. sp. *lycopersici*	Naing et al., 2015.
	*Rhizobium*	*Rhizobium japonicum*	*Fusarium solani, Macrophomina phaseolina*	Al-Ani et al., 2012.
	*Stenotrophomonas*	*Stenotrophomonas maltophilia*	*Ralstonia solanacearum*	Messiha et al., 2007; Elhalag et al., 2015.

Concerning bacterial communities, the OTUs detected in soils C and M also differed from those of soils S and IS, with dominant *Alpha-Proteobacteria* and *Gamma-Proteobacteria* in soils C and M, and dominant *Actinobacteria* in soils S and IS (Figure 5). More precisely, several OTUs affiliated to known bacterial genera were more abundant in soils S than in soil C (*P* < 0.05), *e.g., Adhaeribacter, Amycolatopsis, Arthrobacter, Geobacter, Massilia, Microvirga, Paenibacillus, Rhizobacter, Rhizobium, Rubrobacter*, and *Stenotrophomonas* (Figure 5, underlined OTUs). Interestingly, some of these OTUs (*i.e., Adhaeribacter, Amycolatopsis, Arthrobacter, Paenibacillus, Rhizobacter, Rubrobacter*, and *Stenotrophomonas*) were also more abundant in soil IS than in soil C (*P* < 0.05). Finally, some of these OTUs (*i.e., Adhaeribacter, Amycolatopsis, Paenibacillus, Rhizobacter*, and *Rubrobacter*) were also more abundant in soil M than in soil C (*P* < 0.05). Interestingly, four genera mentioned above (*Arthrobacter, Paenibacillus, Rhizobium*, and *Stenotrophomonas*) are known to have pathogen suppression potential (Table 2), and two of them (*Paenibacillus* and *Rhizobium*) include strains used in the biological control of pathogenic *Fusarium* (Table 2).

## Discussion

Châteaurenard soil has been so far a unique model for understanding the microbial nature of soils naturally suppressive to Fusarium wilts (Alabouvette, 1986, 1999). The uniqueness of this soil lies in the native and long-lasting character of disease suppressiveness, whereas suppressiveness of other soils and other diseases is acquired and transient (Weller et al., 2002; Mazzola, 2007; Mendes et al., 2011). In most cases, attention was mainly paid to decipher the role of bacterial communities (Kyselková et al., 2009; Mendes et al., 2011; Gómez Expósito et al., 2017) while in the present study, both bacterial and fungal communities are taken into consideration. Our suppressive and conducive soils subjected to globally equivalent intensive cropping systems (market gardening) under the same pedoclimatic conditions harbored similar specific bacterial richness. Moreover, diversity indices did not at all highlight specific traits likely to explain the different behaviors with respect to disease. Only fungal communities slightly varied, based on OTU numbers and Shannon and Evenness indices. The highest number of OTUs recorded in the conducive soil amended with suppressive soil may have resulted from the accumulation of specific microorganisms from both soils. Analyses of assigned OTUs showed that bacterial community and even more markedly fungal community membership and structure differed in this Châteaurenard suppressive soil as compared to the nearby conducive soil. Some of these differing OTUs belonged to known genera that harbored strains with biocontrol activity against plant pathogenic fungi including *F. oxysporum*, like *Acremonium, Chaetomium, Clonostachys, Fusarium*, and *Penicillium*. Analyzing the diversity of microbial communities remains descriptive, nevertheless, the presence of these taxa in the suppressive soil of Châteaurenard cannot leave indifferent as for their possible contribution to the control of *F. oxysporum*. For example, the presence and abundance of the *Acremonium* and *Chaetomium* genera were associated with reduced disease and reduced *F. oxysporum* abundance during field observations following specific agricultural practices (Zhao et al., 2014; Huang et al., 2015; Ma et al., 2015). These two genera, along with the *Penicillium* genus, had a similar effect on cereal diseases caused by the Fusarium Head Blight species complex in which *F. oxysporum* is however little involved (Vujanovic et al., 2012; Kohl et al., 2015). Further evidence of the involvement of these genera in *F. oxysporum* control was highlighted under controlled conditions. For example, an *A. chrysogenum* strain controlled *F. oxysporum* f. sp. *melonis* development and reduced disease severity in dual conditions, in vermiculite (Suarez-Estrella et al., 2013). These were analytical studies that revealed a huge diversity of mechanisms within genera, such as mentioned above, that confer an important bioprotective potential against numerous phytopathogenic targets including *F. oxysporum* (Daguerre et al., 2016). These mechanisms are mainly based on the production of secondary metabolites. In the *Chaetomium* genus, and more specifically in the *C. globosum* species, strains produce polyketides such as chaetoviridin A or chaetoglobosin X, which inhibit *F. oxysporum* development (Park et al., 2005; Wang et al., 2012). *Penicillium chrysogenum* strains produce small cysteine-rich fungicidal proteins that target several fungal species including *F. oxysporum* (Kaiserer et al., 2003). *Clonostachys catenulatum* and *C. rosea* are more known to inhibit *F. graminearum* development by producing lactonohydrolases or hydrophobins (Dubey et al., 2014; Popiel et al., 2014), but their ability to control *F. oxysporum* development by producing β-1,3-glucanases besides mycoparasitism has also been demonstrated (Chatterton and Punja, 2009; Tian et al., 2014). Finally, inside the *F. oxysporum* species itself, in addition to the already mentioned trophic competition mechanisms (Alabouvette et al., 2009), strains produce secondary metabolites such as terpenes. These specifically impacted *formae speciales* such as *F. oxysporum* f. sp. *lactucae*, whose growth was inhibited by α-humulene from strain *F. oxysporum* MSA35 (Minerdi et al., 2009). On the other hand, *F. oxysporum* has developed mechanisms of resistance to mycotoxins produced by other species from the *Fusarium* genus (Dawidziuk et al., 2016). Therefore, these other species probably contribute only weakly to soil suppressiveness of Fusarium wilt, or do so via other modes of action which remain to be identified.

None of the few examples of antagonistic activity mentioned above is highly specific or completely biocidal and inhibitory of *F. oxysporum* infectious activity, or permanently active. On the other hand, the combination of these multiple mechanisms most probably exerts a permanent pressure that only allows the pathogen to survive; *F. oxysporum* is still present in Châteaurenard soil, it has not been eradicated. However, due to the multi-factorial nature of this pressure linked to the diversity of mechanisms affecting its metabolism, *F. oxysporum* has not as yet developed means to circumvent this pressure, despite the remarkable adaptability that it is otherwise able to express in various abiotic conditions (Steinberg et al., 2016). This could explain why its pathogenic activity remains controlled in the suppressive soil of Châteaurenard.

Taxa were also found in studies aimed at identifying bacteria associated with soil suppressiveness to Fusarium wilt (Shen et al., 2014, 2015) or with agricultural practices reducing the infectious activity of pathogenic *F. oxysporum* (Klein et al., 2013; Naing et al., 2015). This was particularly the case of *Paenibacillus, Arthrobacter*, and *Rhizobium*. The co-occurrence of these bacteria with disease suppression does not prove that they are necessarily involved in the control of pathogenic *F. oxysporum*. However, hypotheses can be advanced about potential roles they may have since bacteria of the genera highlighted in our analysis have been described for their ability to produce metabolites that can directly or indirectly affect the metabolism of *F. oxysporum*. Thus, bacteria of the *Paenibacillus* genus produce secondary metabolites such as fusaricidins A-D which are specifically toxic to certain fungi including *F. oxysporum* (Mousa and Raizada, 2015). *Actinobacteria* are the most prolific production source of bioactive metabolites, among which various polyketides– antifungal compounds–thanks to many secondary metabolite gene clusters in their genomes (Hamedi and Mohammadipanah, 2015). By the way, they are more abundant in a soil suppressive to Rhizoctonia damping-off of sugarbeet than in conducive soils (Mendes et al., 2011). *Streptomyces* spp. strains produce molecules that inhibit *F. oxysporum* development (Cha et al., 2016). The co-occurrence of *Rhizobium* with disease suppression was *a priori* more difficult to explain. However, *Rhizobium* isolates inhibited *F. oxysporum* f. sp. *ciceris* growth by producing volatiles, mainly cyanides. Although they did not totally control the pathogen in the field, they contributed to decreasing disease incidence (Arfaoui et al., 2006). It may seem strange that OTUs referring to the *Pseudomonas* genus should not be included in the list of bacterial taxa specifically present in the suppressive soil of Châteaurenard, while Lemanceau and Alabouvette (1993) and Mazurier et al. (2009) isolated fluorescent *Pseudomonas* and showed that these strains indirectly reinforced trophic competition between pathogenic and non-pathogenic *F. oxysporum* by reducing Fe availability in the rhizosphere or producing antifungal phenazines. However, the *Pseudomonas* genus is ubiquitous and was indeed detected in all soils, but given their discrimination level due to the length of obtained reads, OTUs did not distinguish the *Pseudomonas* strains directly involved.

The suppressive character of Châteaurenard soil is not only due to the presence of non-pathogenic *F. oxysporum* and *Pseudomonas fluorescens* producing siderophores even though the role of these microorganisms was previously highlighted a lot (Alabouvette, 1986; Lemanceau et al., 1993). Since this suppressive character has been observed for years, that it is stable, it results more likely from a combination of complementary mechanisms of fungal and bacterial origin, besides the role of the abiotic components (pH, clays type) which, although it has not been approached here, also participate in the microbiological functioning of the soil (Höper et al., 1995; Almario et al., 2013a,b). Thus, the sum of the mechanisms expressed concurrently or successively would constrain *F. oxysporum* development and explain the suppressive nature of Châteaurenard soil. *F. oxysporum* survived but did not express its infectious activity. This also explains why Châteaurenard soil is suppressive to Fusarium wilts and not to diseases caused by pathogens with different ecological requirements. These mechanisms are driven by consortiums that include microorganisms directly involved in *F. oxysporum* control; but they are also very likely driven by microorganisms rather believed to act on compatibility and communication between these biocontrol agents, which structure the community assemblage and give the consortium a synergistic value. Analyzing co-occurrence networks within suppressive and conducive soils microbial communities would test such hypothesis (Faust et al., 2015; Karimi et al., 2017).

The taxa revealed by the OTUs are generally presented as genera, but they more often correspond to a sum of individuals whose biological functionality or role may not be detectable at the genus level, but rather at the strain level (Mendes et al., 2011). It is therefore difficult to assign a direct or indirect antagonistic activity to these OTUs against *F. oxysporum*. Nevertheless, we found them associated with suppressive soil, and they were associated with Fusarium disease decline in other studies. Consequently, we may believe that certain strains from these taxa are actually involved in the mechanisms of soil suppressiveness. To confirm this hypothesis, it would be necessary to isolate these microorganisms by conventional methods, insofar as Pasteurian methods allow for it, which is not always the case. However, intrageneric or even intraspecific diversity is such within these taxa that the number of isolated microorganisms should be exhaustive to detect the bioactive strain(s) actually involved in the control of pathogenic *F. oxysporum*. Additionally, there is no evidence that any given strain taken separately can exert its antagonistic activity in the same way as when it is within the microbial community. On the contrary, a better efficiency of the biological control agents is obtained when microbial consortia and not single strains are used to control pathogenic fungi in soils and substrates (Danon et al., 2010; Jain et al., 2012; Pertot et al., 2017). Thus, it is becoming clearer and clearer that soil functionality depends on the community pattern and that the activity of the microorganisms directly involved in the targeted function is only possible in the presence of the different components of this community, without these components being directly involved in the function of interest (Tyc et al., 2014; Williams et al., 2014; Chao et al., 2016). Therefore, an alternative strategy to identify the members of the microbial consortium responsible for soil suppressiveness would be to erode diversity by performing serial dilutions of the suppressive soil until it loses its suppressiveness. The dilution level preceding the loss of suppressiveness would contain the minimum consortium necessary for pathogenic *F. oxysporum* control. This erosion of diversity and/or destructuring of the community pattern may well be responsible for the loss of the suppressive character of soil plots in the Châteaurenard region. Actually, the soil is suppressive to Fusarium wilts but not to other soil-borne diseases. In market gardening, producers use disinfection methods (fumigation or/and steam) to control other soil-borne diseases such as damping-off during rotations (Navarrete et al., 2006). Consequently, the repeated use of these practices strongly and more or less permanently disrupted the microbial balance to reach a similar situation to the one in our comparative study. Other farming practices such as intensive monoculture also led to the accumulation of pathogens and reduced the number and/or activity of beneficial bacteria, with a loss of soil suppressiveness to cotton Fusarium wilt (Li et al., 2015). Conversely, modifying the structure of microbial soil communities through agricultural practices can make soils temporarily suppressive to one disease or another (Westphal and Becker, 2001; Mazzola, 2007; Klein et al., 2013; Raaijmakers and Mazzola, 2016; Vida et al., 2016). Unfortunately the sustainability of this suppressiveness seems difficult to acquire as durably as in Châteaurenard soil. It is therefore likely that the balance reached by Châteaurenard soil microbial communities results from a long natural evolutionary process; the suppressiveness of this soil is called “native,” this is the reason why it is so stable. In the cases of “acquired” soil suppressiveness, the agricultural practices used to manage microbial community patterns are still too recent to fix their assemblage (structure) in a sustainable way, but all the results reported in the literature show that this is an alternative to be favored.

## Author contributions

The project was supervised by CS. KS-H, VE-H, and EC: carried out the experiments and acquired the data; VE-H and ST: carried out bioinformatics analyzes; VE-H, ST, JR, and CS: animated scientific discussions over the duration of the project and contributed equally to the writing of the article.

### Conflict of interest statement

The authors declare that the research was conducted in the absence of any commercial or financial relationships that could be construed as a potential conflict of interest.
