# Supplementary material for: Comparative Microbiome Analysis of a Fusarium Wilt Suppressive Soil and a Fusarium Wilt Conducive Soil From the Châteaurenard Region
Source: Front Microbiol. 2018 Apr 4;9:568. doi: 10.3389/fmicb.2018.00568 (PMC5893819; doi:10.3389/fmicb.2018.00568)
Supplement: Supplementary file 2 [file DataSheet1.docx]

**SUPPLEMENTARY FIGURE 1.** Rarefaction curves of fungal (A) and bacterial (B) OTUs detected in the conducive soil (C), mixed soil (M), suppressive soil (S) and pathogen-inoculated suppressive soil (IS).

A
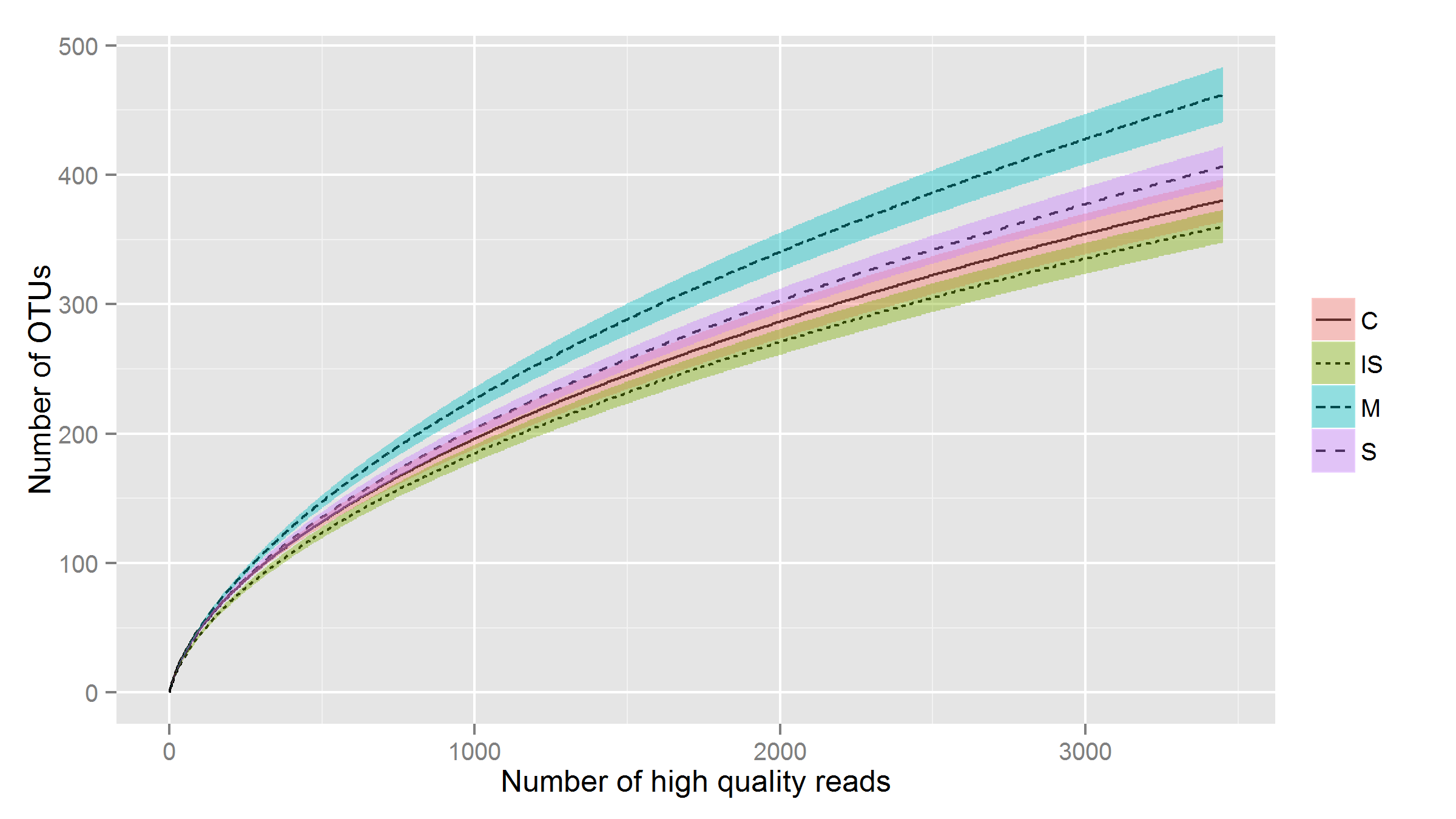


**B
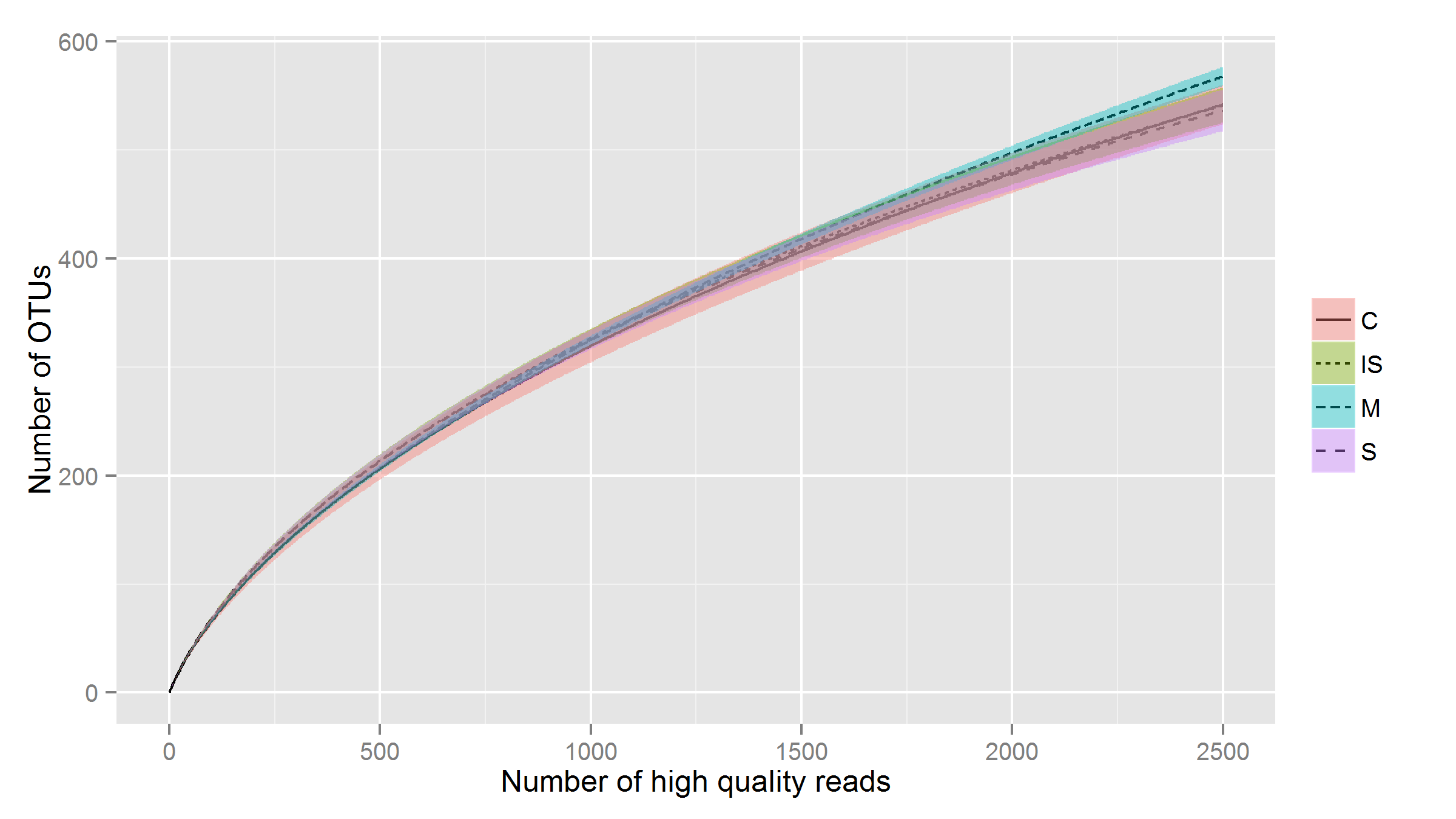
**
